# Short chain fatty acids facilitate protective immunity by macrophages and T cells during acute fowl adenovirus-4 infection

**DOI:** 10.1038/s41598-023-45340-8

**Published:** 2023-10-21

**Authors:** Rangyeon Lee, Byung-Il Yoon, Christopher A. Hunter, Hyuk Moo Kwon, Haan Woo Sung, Jeongho Park

**Affiliations:** 1 College of Veterinary Medicine, Kangwon National University, Chuncheon, Republic of Korea; 2 Institute of Veterinary Science, Kangwon National University, Chuncheon, Republic of Korea; 3 School of Veterinary Medicine, University of Pennsylvania, Philadelphia, USA; 4 Multidimensional Genomics Research Center, Kangwon National University, Chuncheon, Republic of Korea

**Keywords:** Adaptive immunity, Infection, Infectious diseases, Adenovirus, Virus-host interactions, Infectious diseases, Immunology, Microbiology

## Abstract

Short chain fatty acids (SCFAs) are major gut metabolites that are involved in the regulation of dysfunction in immune responses, such as autoimmunity and cytokine storm. Numerous studies have reported a protective action of SCFAs against infectious diseases. This study investigated whether SCFAs have protective effect for immunity during fowl adenovirus-4 (FAdV-4) infection. We examined whether SCFA mixture (acetate, propionate, and butyrate) administration could protect against intramuscular challenge of a virulent viral strain. SCFA treatment promoted MHCII-expressing monocytes, the active form of T cells, and effector molecules in both peripheral and lymphoid tissues. It also boosted the production of immune molecules involved in pathogen elimination by intraepithelial lymphocytes and changed the intestinal microbial composition. We suggest that gut metabolites influence the gut microbial environment, and these changes stimulate macrophages and T cells to fight against the intramuscular challenge of FAdV-4.

## Introduction

Fowl adenovirus (FAdV) is a non-enveloped dsDNA virus that belongs to the *Aviadenoviridae* family and *Adenovirus* genus^1,2^. As one of 12 serotypes, infection by FAdV-4 causes lethal diseases such as hepatitis-hydropericardium (HHS) and inclusion body hepatitis (IBS)^3,4^. This is a highly contagious pathogen that is fatal to 3–5-weeks-old chicks^5,6^, and global outbreaks of FAdV-induced diseases provoke massive economic loss, which requires the development of new preventive strategy^7^.

During the FAdV-4 infection, innate immune cells preferentially recognize the pathogen by pattern recognition receptors (PRRs) including toll-like receptors (TLRs) and NOD-like receptors (NLRs). The activation of PRRs initiates cytokine and chemokine production through intracellular signaling pathways such as NF-κB, and Myd88. The resulting immune processes stimulate both innate and adaptive immunity^8–10^. For example, the release of effector molecules including TNF-α, IFN-γ, and IL-1β, and the regulatory cytokine, IL-10 are stimulated by this virus, which also modulates the expansion of lymphocytes^6,11^. Although the pathogenesis of and immune responses to adenovirus infection have been studied in other animals, there are few reports on the immune responses in birds.

Among the various gut metabolites, short chain fatty acids (SCFAs) are a major product of non-digestible polysaccharide fermentation. Most SCFAs comprise acetate, propionate, and butyrate and at 10 ~ 100 mM in the intestines^12^. The effects of acetate, propionate, and butyrate are not limited to the gastrointestinal tract (GIT), and these molecules are involved in the regulation of immune responses in other peripheral organs. SCFAs act through several mechanisms: G-coupled receptor (GPR) signaling, histone deacetylase (HDAC) inhibition, and metabolic pathways such as glycolysis and acetyl-CoA production. The gut metabolites eventually orchestrate adaptive immunity by managing T cell differentiation and cytokine production^13–16^. For example, acetate, propionate, and butyrate promote naïve T cell differentiation into Th1 and Th17 cells. They also induce regulatory T cells that secret IL-10, which prevents excessive inflammation, and cytotoxicity and the recall responses of CD8^+^ T cells were enhanced^17–20^.

During infection, intestinal microbiota facilitate protective immunity by stimulating the production of cytokines and anti-microbial molecules^21^. Invading pathogens can modulate the microbial community and its production of acetate, propionate, and butyrate^22^, and acetate treatment facilitates antiviral actions. For example, during influenza infection, SCFAs induce patrolling monocytes in the lung by activating FFAR3, macrophages involved in tissue regeneration, and cytotoxic T cells are induced^22,23^. Acetate (C2)-mediated IFN-β induction facilitates immunity to respiratory syncytial virus (RSV) infection^24^. Butyrate (C4)-induced transcriptional changes in macrophages strengthen bactericidal activity^25^.

The protective actions of SCFAs have been reported in various animal models. In food animals such as chickens, beneficial actions of SCFAs include anti-inflammation action and increased body weight^26^, and the chicken industry uses SCFAs as a feed additive^27–29^. However few studies have reported on whether SCFAs afford protective immunity in chickens. This study analyzed the effect of SCFAs mixture (acetate, propionate, and butyrate) administration on the responses of avian immune cells to viral infections. We examined both innate and adaptive immune cell regulation during FAdV-4 infection and the participation of the gut microbiota and its metabolites in protective immunity.

## Results

### Protection against FAdV-4 infection mediated by SCFA administration

**Figure 1:**
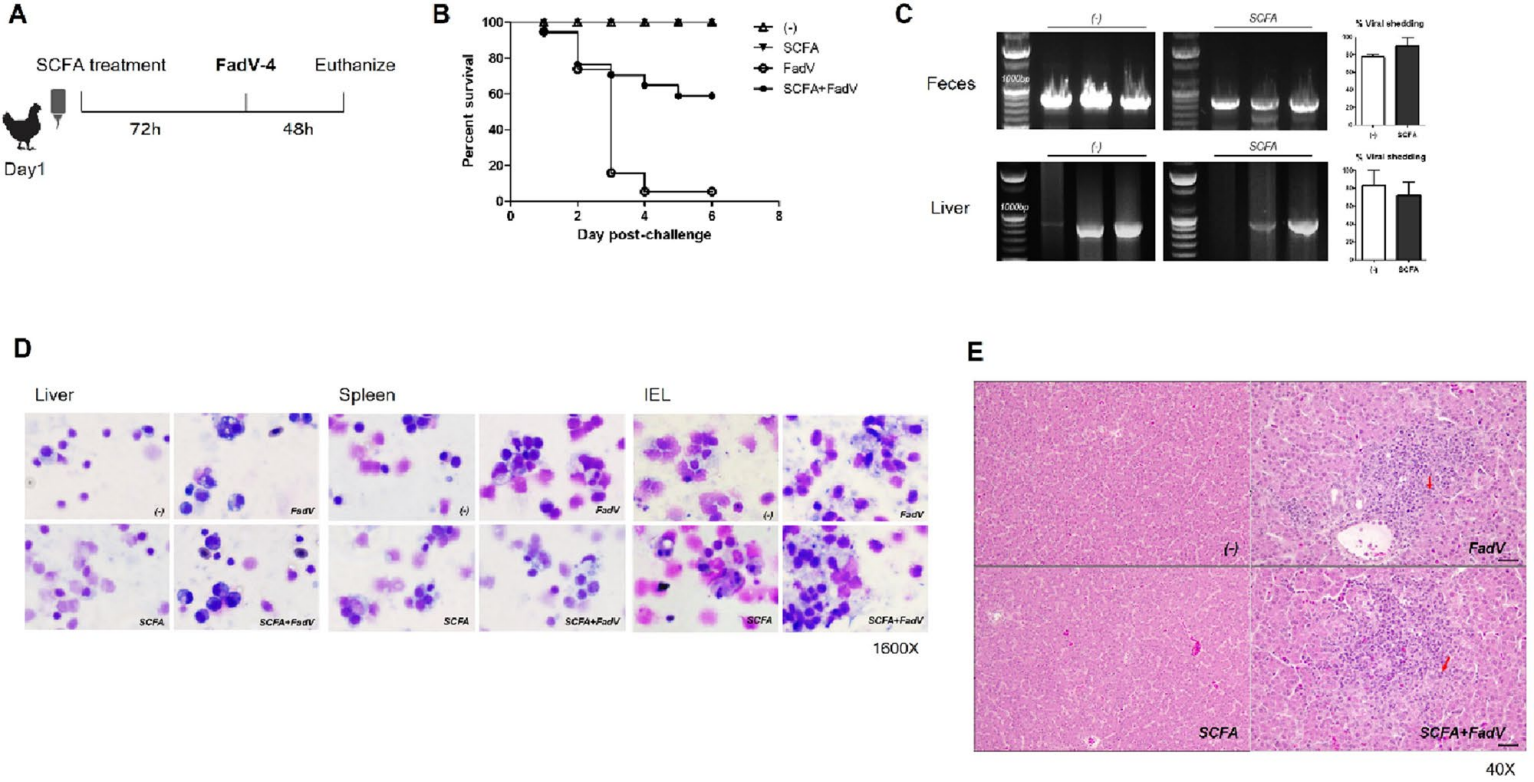
Effect of short chain fatty acids (SCFAs) on survival rate, viral clearance, and cytology. (**A**) Chickens (4 days old) treated with or without SCFA water were injected intramuscularly with FAdV-4 or PBS. (**B**) The survival rate was calculated after challenge with FAdV-4. Six chickens in uninfected groups (3 normal water-fed and 3 SCFA-fed) and 18 animals in infected groups (9 FAdV and 9 SCFA + FAdV) from two individual experiments. The experiments were repeated in triplicate, and pooled data are shown. (**C**) The viral gene was determined by PCR in feces and livers from infection group (Feces n = 3 ~ 4, Liver n = 2 ~ 3 per group) At least two independent experiments were performed, and the pooled data are shown. (**D**) Representative Cytospin images of splenocytes, liver cells, and intraepithelial lymphocytes (IELs) at 2 dpi are shown. (**E**) Histological changes in liver at 2dpi from each group are shown. Arrows indicate intranuclear inclusion bodies.

After SCFA pre-treatment for 72 h, chickens were infected with a virulent FAdV-4 strain. FAdV-4 infection develops acute and fatal hepatic inflammation. Within five days post infection, chickens showed drastic increase in liver enzyme and hepatic apoptosis, which is accompanied by inflammatory molecule expression. Those clinical indexes peaked around at one week post infection and subsided afterwards. In line with previous studies, most untreated animals were not survived around 4 days post-infection (dpi)^6,30^. By contrast, up to 80% of SCFA-treated chickens survived the viral infection (Fig. 1A and B). We next examined the residual virus in each group and found that SCFA feeding did not significantly change the viral detection rate in both liver and fecal material (Fig. 1C). Because we hypothesized that SCFA treatment affects protective immunity, the morphology of cells on cytospins from the liver, spleen, and IELs was examined. Some activated lymphocytes were detected, but their structure did not differ much between groups (Fig. 1D). Histopathological examination showed focal necrosis and inflammatory foci were typically observed in the livers of the virus-infected chicks, characterized by infiltration of mononuclear cells and/or heterophils with hepatocytic vacuolar degeneration and necrosis. In the lesions, intranuclear basophilic inclusion bodies were evident in some hepatocytes, demonstrating that the inflammatory lesions were induced by the adenovirus infection. However, the morphological characteristics of the inflammatory lesions and its severity were similar in the virus infected groups with or without SCFA treatment (Fig. 1E and Table 1). Given the action of SCFA administration observed, we next examined whether this is related to regulation of the immune system.

**Table 1:** Histopathological examination in the liver.

Group	No treatment + no infection	No treatment + infection	SCFA + no infection	SCFA + infection
No. examined	3	3	3	3
No specific lesion	2 (66.7)	0 (0.00)	1 (33.3)	0 (0.00)
Infiltrates, mononuclear cells, multifocal	1 (33.3)	2 (66.7)	1 (33.3)	2 (66.7)
*Grades: minimal*	1	0\	0	0
*Mild*	0	2	1	2
Focal necrosis, multifocal	0 (0.00)	2 (66.7)	0 (0.00)	0 (0.00)
*Grades: minimal*	0	2	0	0
Inflammatory foci, heterophilic and/or mononuclear cells with vacuolar degeneration and necrosis of hepatocytes	0 (0.00)	3 (100)	0(0.00)	3 (100)
*Grades: moderate*	0	3	0	3
Lipidosis, diffuse	1 (33.3)	2 (66.7)	1 (33.3)	2 (66.7)
*Grades: minimal*	1	1	1	2
*Mild*	0	1	0	0

### Effect of SCFA and infection on immune cell phenotype and function in the target organs

**Figure 2:**
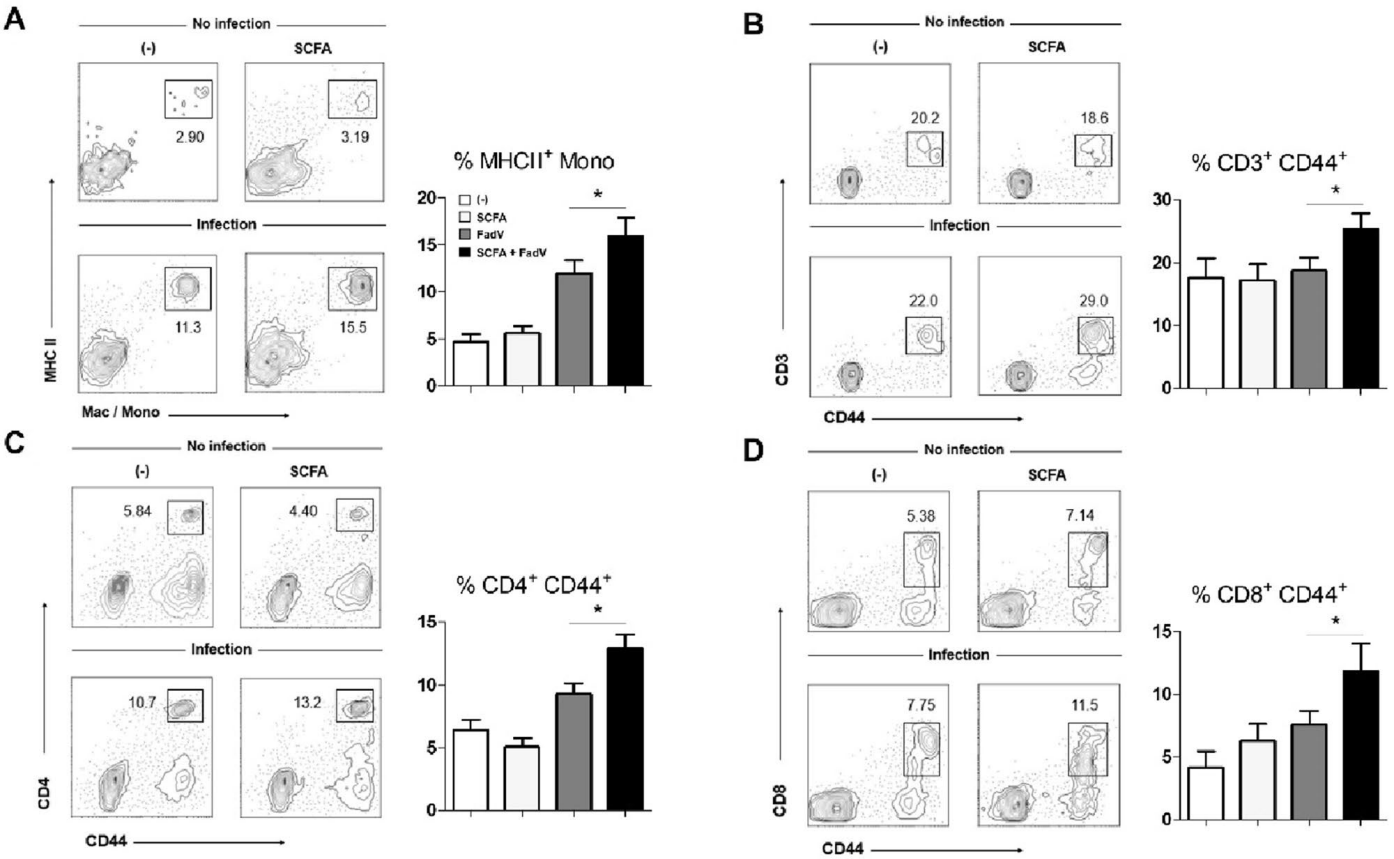
Effects of SCFAs on immune cell subsets in the liver during FAdV-4 infection. The frequencies of macrophages/monocytes and T cell subsets in liver were determined using flow cytometry at 2 dpi. (**A**) MHCII^+^ macrophages/monocytes were examined. Representative plots are shown. (**B**–**D**) Frequencies of CD44-expressing T cells (CD3^+^, CD4^+^, and CD8^+^) were calculated and representative plots are shown. Cells were obtained from 3 to 5 chickens per group. At least three independent experiments were performed, and pooled data are shown. Significant differences were identified using one way ANOVA. (*p < 0.05).

After observing the effect of the SCFA supplementation against viral infection, we next examined the response of the liver, because this is the target organ for FAdV4 infection and acute infection causes hepatic pathology^6^. We focused on immune cells associated with protective immunity in the liver. The hepatic population of activated monocytes was examined by analyzing MHCII expression on their surfaces. Before infection, the MHCII-expressing myeloid cell frequency was less than 5% and the statistical significance (*p* < 0.05) was not observed between groups. However, the monocyte population expanded following infection and even further after SCFA treatment (Fig. 2A). The contact between hepatic resident macrophages, such as Kupffer cells, and invading pathogens initiates phagocytosis^31^. Because hepatic myeloid cells regulate adaptive immunity, we investigated T and B cell populations. Most hepatic CD3^+^ T cells exist in the activated form that expresses CD44, and the CD44^+^ population was significantly higher in the SCFA-treated group (Fig. 2B). CD44-expressing CD4^+^ and CD8^+^ T cell populations were also expanded by SCFA treatment during viral infection (Fig. 2C and D). We also examined γδ-T cells and B cells in liver during viral infection. A few more γδ-T cells were detected in the SCFA-treated group, but the B cell population was unchanged (S. Figure 1).

**Figure 3:**
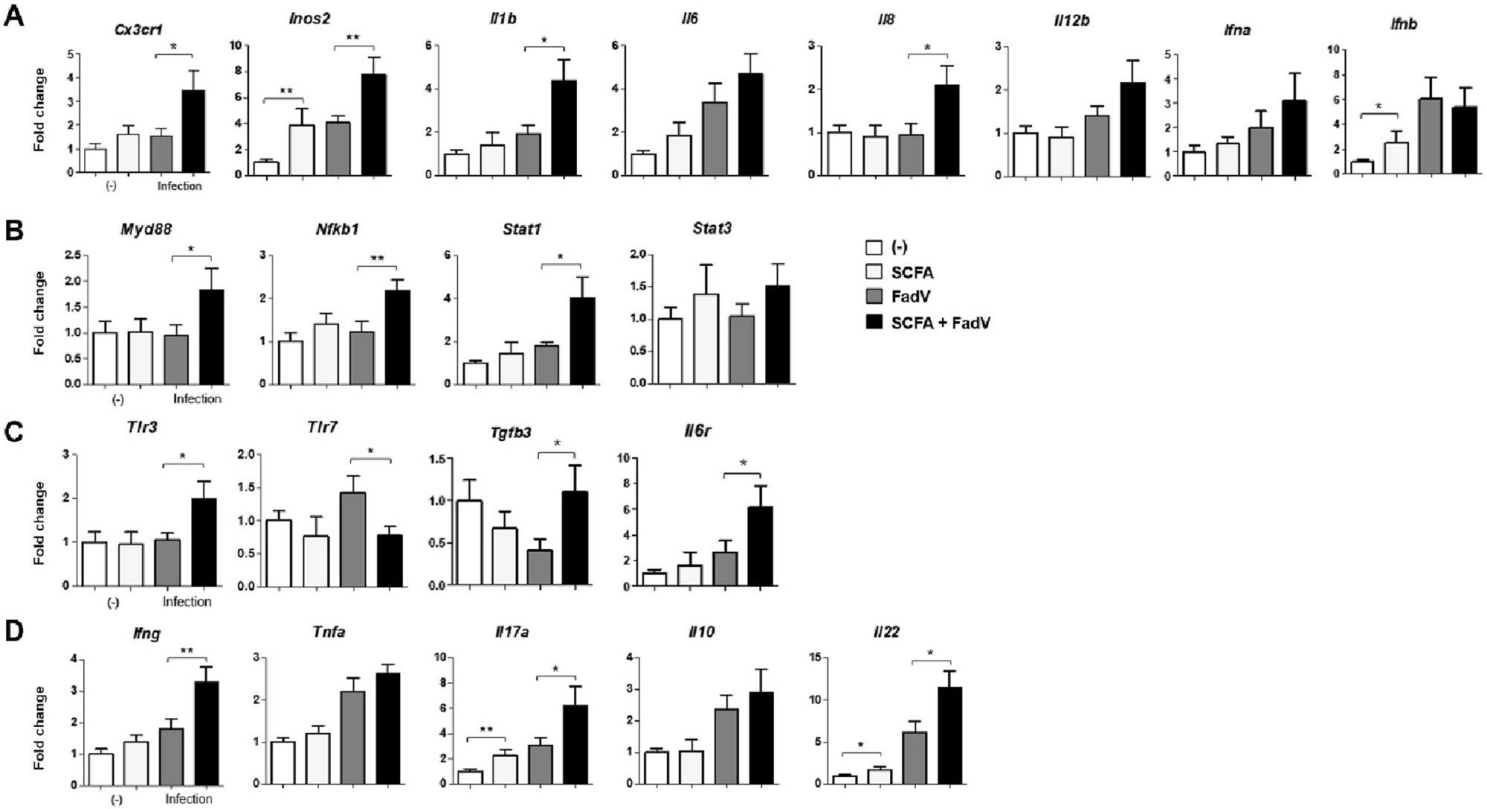
mRNA expressions of immune-related genes in the liver. Levels of mRNA for immune-related molecules in the liver tissue examined using quantitative PCR. (**A**) Macrophage-associated cytokine genes (*cx3cr1, inos2, il1b, il6, il8*, and *il12b*) and type1 interferon genes were analyzed. (B) T cell cytokine genes (*ifng, il10, il17a,* and *il22*) (**C**) Expressions of genes for Toll-like receptors (*tlr3 and tlr7*) are shown. (**D**) Expressions of genes for cell signaling pathway (*myd88, nfkb2, stat1,* and *stat3*) are shown. Liver tissues were collected from 3 to 5 chickens each group at 2 dpi. The data were combined from three independent experiments, and qPCR was conducted in duplicate. The relative expression levels are presented as fold changes compared with the β-actin gene. Significant differences were identified using unpaired t test (*p < 0.05, **P < 0.01).

Next, we analyzed immune functions in tissue-resident cells during FAdV-4 infection. The gene expression levels of genes encoding for effector cytokines and immune-regulating molecules were examined in liver cells. During intracellular infection, CX3CR1^+^ monocytes patrol through vessels in the epithelium^32,33^. We found that, in addition to an increase in the number of MHCII-expressing monocytes noted above, *cx3cr1* expression was upregulated by SCFA administration in infected animals. In this group, the genes for myeloid-derived cytokines such as IL-1β, IL-6, IL-8, and IL-12 were also highly expressed, as were the expression levels of genes for other effector molecules such as iNOS2 and type I cytokines (Fig. 3A).

These macrophage-releasing molecules and intracellular regulators are critical for the generation of antigen-specific T cells^34^. The genes for STAT molecules and NF-κB were expressed at higher level in SCFA-treated group than the control group (Fig. 3B). We think that these increases were related to increased levels of TLR3 and MyD88 (Fig. 3C). We next examined whether SCFA administration increased the expression of the genes for cytokines secreted by T cells such as IFN-γ, TNF-α, and IL-17A. The expression of the genes encoding IL-10 and IL-22 were also coordinated with SCFA treatment (Fig. 3D). Taken together, these data suggest that the major gut metabolites, SCFAs, act as a powerful stimulator of effector immune cells and induce the secretion of specific molecules in the body periphery during FAdV-4 infection.

### Effect of SCFA and infection on immune cell phenotype and function in lymphoid organs

**Figure 4:**
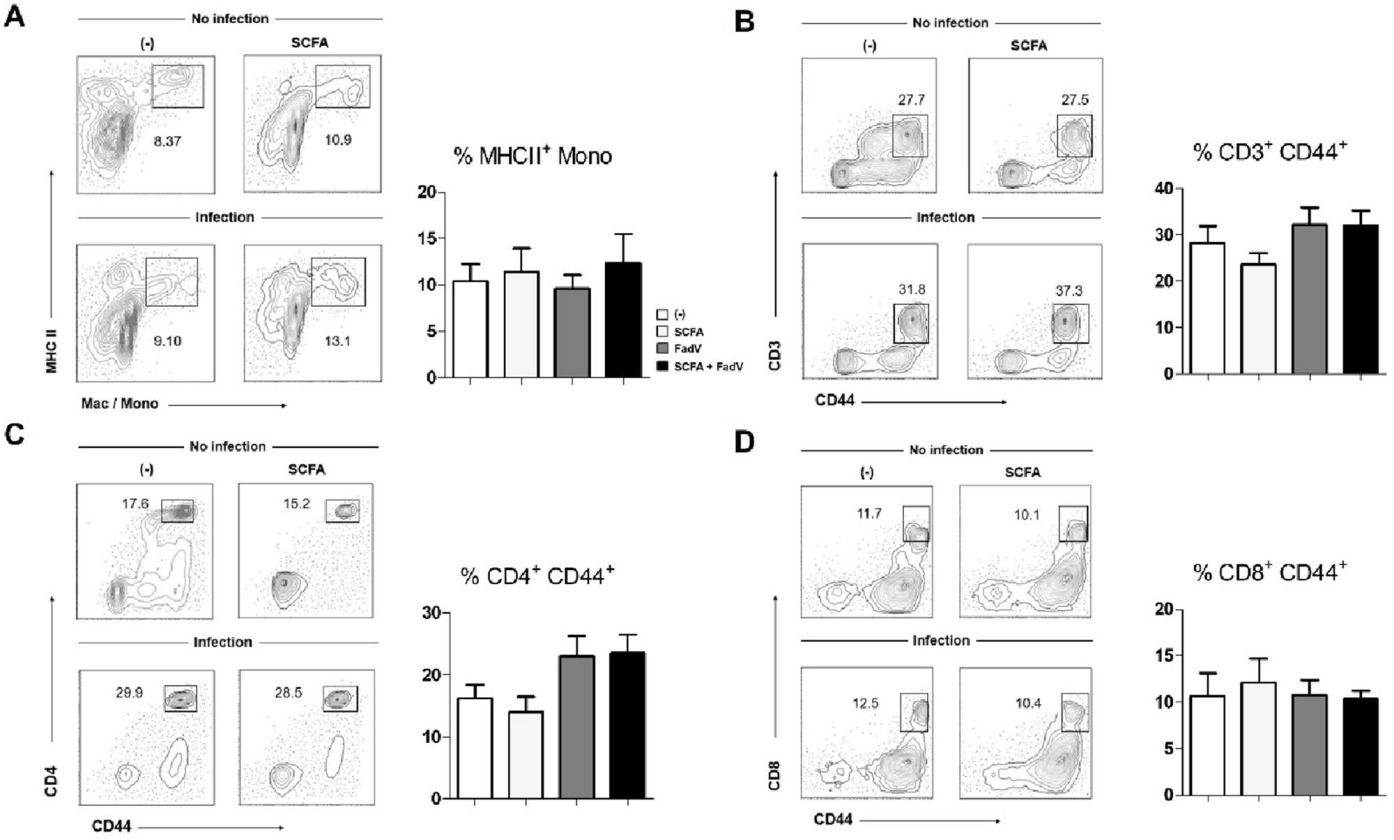
Effects of SCFAs on immune cell subsets from the spleen during FAdV-4 infection. The frequencies of macrophage/monocyte and T cell subsets of splenocytes were determined. (**A**) Frequencies of MHCII^+^ macrophages/monocytes were examined. Representative plots are shown. (**B**–**D**) Frequencies of CD44-expressing T cells (CD3^+^, CD4^+^, and CD8^+^) were calculated and representative plots are shown. Cells were obtained from 3 to 5 chickens per group. At least three independent experiments were repeated, and pooled data are shown. Significant differences were identified using one way ANOVA (*p < 0.05).

Around 5 dpi, the viral antigen is detectable in spleen^35^. We investigated whether exposure to viral antigen affects immune cells in this major lymphoid tissue. MHCII-expressing monocytes were present even before the infection, and this population was not changed by infection or SCFA treatment (Fig. 4A). Infection increased the frequencies of activated CD3^+^ and CD4^+^ T cells, but these frequencies were not increased further by SCFA (Fig. 4B and C). The size of the activated CD8^+^ T cell and B populations were consistent over the course of infection (Fig. 4D and S. Figure 2B). By contrast, the γδ-T cell population was increased significantly by the combination of SCFA and infection (S. Figure 2A).

**Figure 5:**
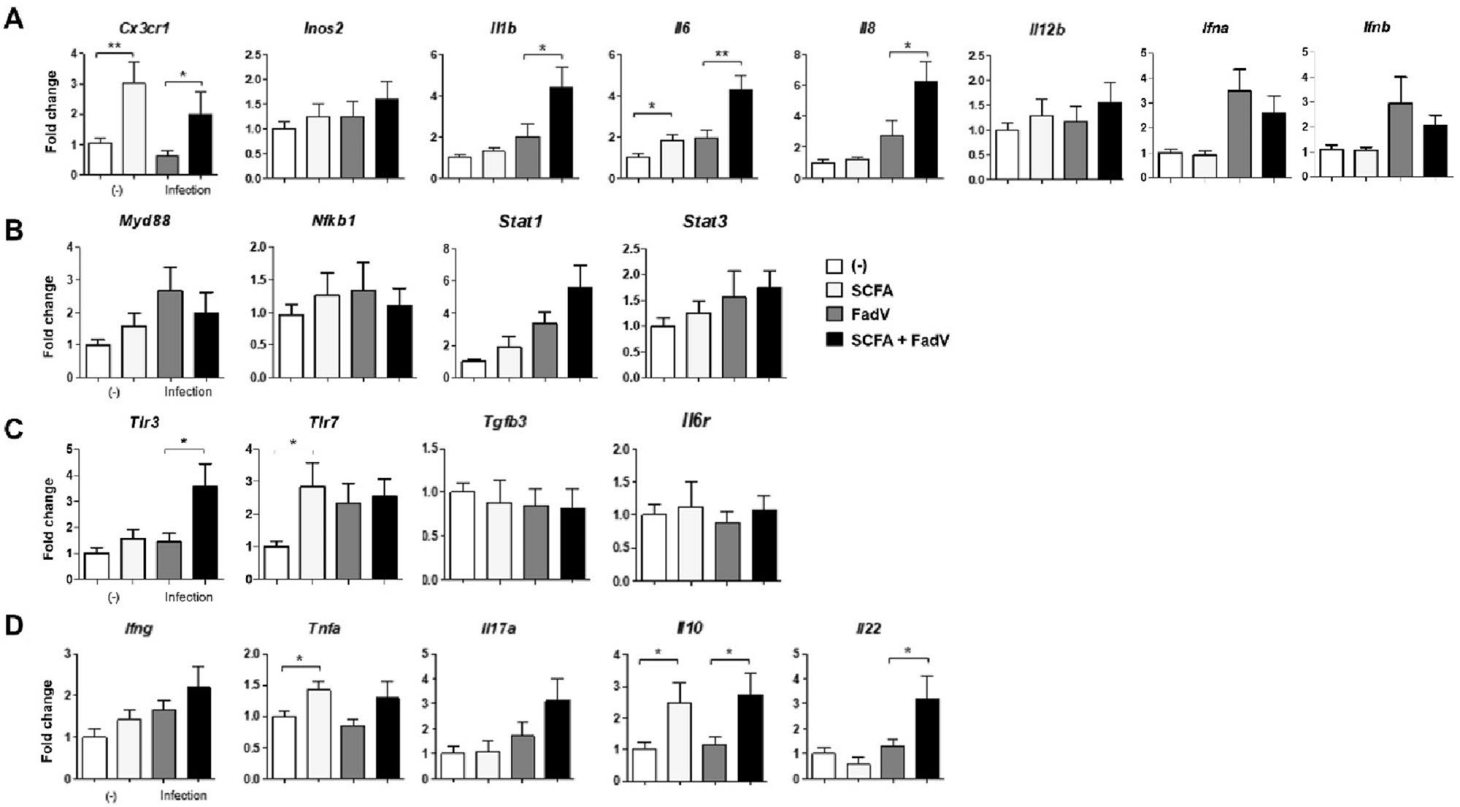
Differences in mRNA expression of immune-related genes in splenocytes. The mRNA levels of immune-related molecules in the splenocytes were examined using quantitative PCR. (**A**) Genes for macrophage-associated cytokines (*cx3cr1, inos2, il1b, il6, il8*, and *il12b*) and type I interferon were analyzed. (**B**) T cell cytokine genes (*ifng, il10, il17a,* and *il22*) were analyzed (**C**) Expressions of genes for Toll-like receptors (*tlr3 and tlr7*) are shown. (**D**) Expression of genes for cell signaling pathway (*myd88, nfkb2, stat1,* and *stat3*) are shown. Liver tissues were collected from 3 to 5 chickens each group at 2 dpi. The data from three independent experiments were combined, and qPCR was conducted in duplicate. The relative expression levels are presented as fold changes compared with the β-actin gene. Significant differences were identified using unpaired t test (*p < 0.05, **P < 0.01).

After assessing the phenotype of immune cells during viral infection, we analyzed the function of splenocytes using the same panels of effector and immune-regulating molecules as used to analyze hepatocytes. The expression of genes for myeloid cell-releasing cytokines including IL-1β, IL-6, and IL-8, except type-I IFNs and iNOS2, were increased in the infected and SCFA-treated group. Of note, *cx3cr1* expression was highly upregulated by SCFA treatment in both infected and uninfected animals (Fig. 5A). Unlike in hepatocytes, the expression of genes for intracellular signaling molecules and innate receptors did not differ between groups, except for *tlr3*, whose expression was activated by the combination of SCFA and FAdV-4 infection (Fig. 5B and C). Among the genes for T cell-mediated factors, the expression of genes for regulatory molecules such as IL-10 and IL-22 was increased by SCFA and infection, but that for other inflammatory molecules was not (Fig. 5D). The analysis of immune function in splenocytes showed partial regulatory action of myeloid and T cells.

### Gut-mediated immune response and microbial change during infection

**Figure 6:**
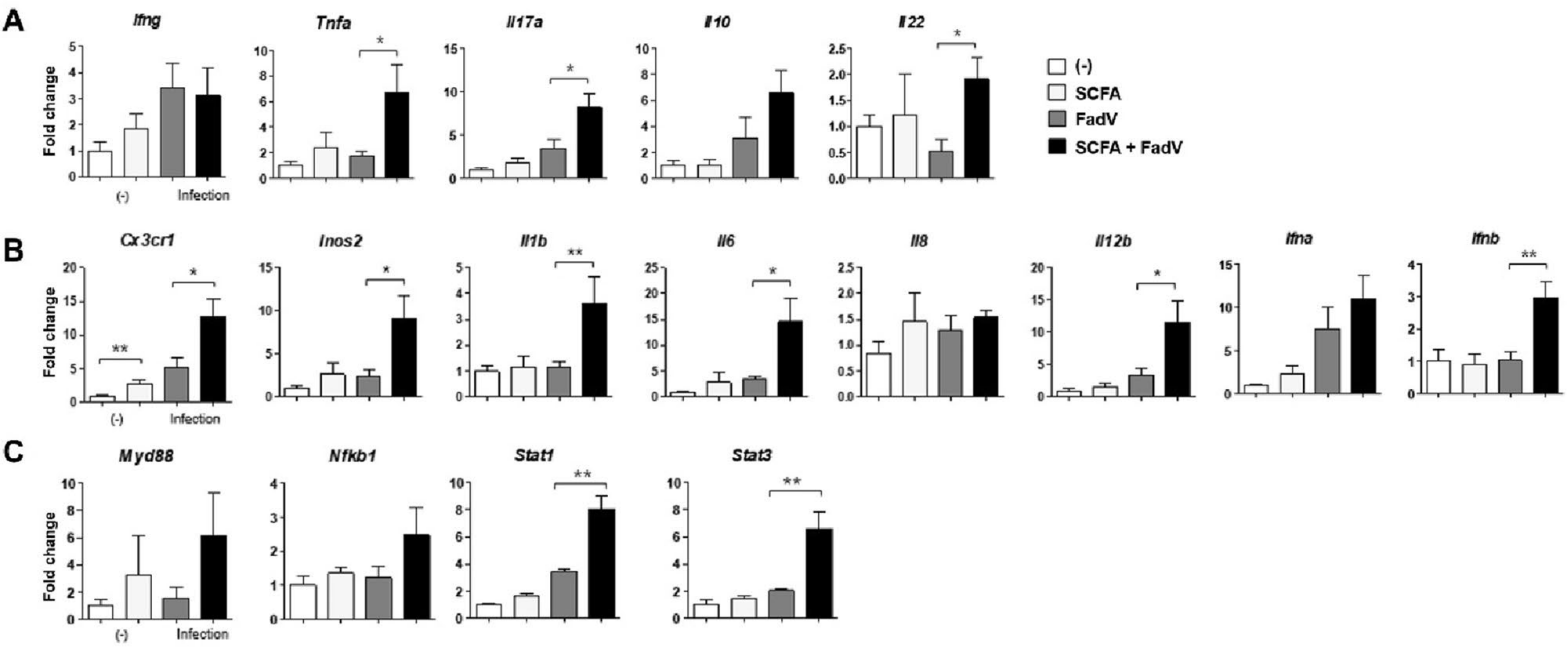
Differences in mRNA expression of immune related genes in IELs. The mRNA levels of immune-related molecules in the IELs were examined through quantitative PCR. (**A**) Genes for macrophage-associated cytokines (*cx3cr1, inos2, il1b, il6, il8*, and *il12b*) and type I interferon were analyzed. (**B**) Genes for T cell cytokines (*ifng, il10, il17a,* and *il22*) are shown. (**C**) Expression of genes for cell signaling pathways (*myd88, nfkb2, stat1,* and *stat3*) are shown. Liver tissues were collected from 3 to 5 chickens from each group at 2 dpi. The data from three independent experiments were combined, and qPCR was conducted in duplicate. The relative expression levels are presented as fold changes compared with the β-actin gene. Significant differences were identified using unpaired t test (*p < 0.05, **P < 0.01).

IELs are located within the intestinal lumen where exogenous antigen and gut metabolites are present. Both αβ- and γδ-T cells are found in IELs that are involved in the pathogenesis of inflammatory disorders and infections^36,37^. We examined whether SCFA treatment facilitates expressions of genes for T cell cytokines in the intestines. Genes for both effector and regulatory cytokines in IELs were upregulated during infection in SCFA-treated group (Fig. 6A). In the infected animals, genes for effector molecules in myeloid cells and type-I IFNs were also activated markedly by SCFA administration (Fig. 6B). Among intracellular signaling molecules, genes for NF-κB, STAT1, and STAT3 were more expressed in SCFA-fed group than the control group (Fig. 6C) We assume that the regulation of gene for T cell cytokines was reflected in the stimulation of myeloid cells in IELs.

Microbial composition in the intestines regulates immune responses and this can be modified by infection and SCFA feeding^38,39^. We examined the proportions of microbial genes in fecal material and compared these between group. In uninfected animals, SCFA administration partially enriched for the genes for *Actinobacteria* and *Bacteroidetes*. By contrast, in uninfected animals, SCFA promoted the expression of genes for *Firmicutes* and *Enterobacteriaceae*. However, neither infection nor SCFA treatment significantly affected the microbial population (S. Figure 3). These results suggest that SCFA shapes, to some extent, the balanced immune responses and regulates microbial composition in the GIT.

## Discussion

SCFAs (acetate, propionate, and butyrate) are major gut metabolites that participate in the regulation of immune responses to various inflammatory pathogenesis including acute infection^12,17,39^. Numerous types of viruses and their variants have a huge economic impact on the poultry industry despite continuous vaccine development^40–43^.

However, it is unknown whether SCFAs contributes to protective immunity during major avian viral infection. In this study, we found that supplementation of acetate, propionate, and butyrate promoted survival of chickens during acute viral infection and that this was accompanied by changes in some immune cells. MHCII-expressing monocytes and activated T cell populations, including αβ- and γδ-T cells, were expanded in the target organs in SCFAs-treated animals. The expressions of genes for effector functions were also promoted in both the liver and spleen, and the action might be closely associated with GIT. Our data suggest that gut metabolites help to regulate cellular immune responses and have antiviral actions during major viral infection in chickens.

Immune-stimulating conditions provide hostile conditions for viral spread. Damage to the endothelium by infection or inflammation stimulates CX3CL1 (fractalkine) expression, which enables CX3CR1^+^ monocytes to adhere to the endothelium in the affected tissue^44^. Considering the increase in *cx3cr1* expression found in our study and the regulation of CXCL1 regulation by SCFAs^23^, we hypothesize that SCFA feeding shaped the generation and migration of patrolling monocytes to the liver during the infection. We also observed genes for myeloid-derived effector cytokines were upregulated markedly in SCFA-treated group. Moreover, SCFA could activate protective action in liver-gut axis. We hypothesize that the gut barrier that protects liver via tight junction might have been strengthened by supplementation of acetate, propionate, and butyrate^45,46^.

Virulent FAdV-4 infection induces acute inflammation and the expression of innate immunity-related genes such as those for IL-1β, IL-6, IL-8, IL-12, iNOS, and TLRs in the spleen. These genes direct antiviral actions of the host by initiating adaptive immunity^47,48^. As in other animals, avian MHCII^+^ splenocytes are induced by inflammation, act as sources of effector cytokine, and exhibit phagocytic and bactericidal activities^49^. SCFAs support the balance between immune responses and stimulating regulatory molecules. SCFA-treated animals have elevated expression of genes for IL-10 and effector cytokines in the spleen. Administration of an high fiber diet (HFD) and SCFAs induced alternative activation profiles in patrolling monocytes, whereas granulocytes and dendritic cells were unaltered^23^. As a result, IL-10 expression in target organs is elevated in FFAR3-dependent manner^17,23,39^. Taken together, these findings imply that SCFA-driven hepatic myeloid cells are patrolling monocytes that are associated with alternative activation profiles.

In addition to producing cytokines, intravascular monocytes express MHCII molecule and present antigens to T cells in peripheral tissues^50^. Expression of immune-regulating genes and MHCII-expressing monocytes increased the most in the group given the combined FAdV-4 infection and SCFA treatment. This finding suggests that SCFAs boosted protective immunity by regulating both myeloid and effector T cell differentiation.

During intracellular infection such as those caused by viruses and protozoan parasitic invasions, the cytotoxic actions of CD8^+^ T cells are critical for the pathogen control. Upon recognition of pathogenic antigens, memory T cells undergo three differentiation steps: central, intermediate, and terminal effector stages. Terminally differentiated effector cells secrete effector molecules such as IFN-γ and TNF-α, but they are short-lived. These cells can be identified by the expression of KLRG1 or CX3CR1 on their surface. The dynamic supply of terminal effector T cells is maintained by the intermediate population, which is proliferative and metabolically active^51,52^.

During the fatal FAdV-4 infection, depletion of lymphocytes in the thymus, spleen, and bursa of Fabricius was examined and the consequent drastic suppression of both CD4^+^ and CD8^+^ T cells were observed. However, the chimeric fiber vaccination stimulated T cell responses and increased lymphocyte population in lymphoid organs^11,53^. Although we were not able to characterize T cell subsets in detail in the chickens because of limited antibody availability, we found that memory CD8^+^ T cells and CX3CR1 expression were promoted by SCFA administration during infection. A recent study reported a pivotal role of gut microbiota and the metabolite, butyrate, during viral infection. When viral antigen was perceived, the gut microbiota and SCFA supported the differentiation of T cell from the naïve to the memory phenotype, which expresses CX3CR1 and CD44. The transition into memory T cells resulted in the production of effector molecules such as IFN-γ in an antigen-specific manner^18^. Consistent with that report, our results also support that SCFA directed the proliferation of intermediated memory T cells, which should lead to terminal differentiation into effector T cells that secret effector molecules such as IFN-γ and TNF-α. Essential intracellular signaling pathways must be activated to induce effector cytokine production. For example, T cell recognition of antigens presented by patrolling monocytes induces NFAT1 translocation to the nucleus, which directs IFN-γ secretion. Activation of the NF-kb, STAT1, and STAT3 intracellular signaling pathways is also critical to the production of effector cytokines during viral infections^50,54,55^. We examined whether SCFA supplementation would increase the expressions genes encoding NF-kb, STAT1 and STAT3, but further studies are needed to identify mechanisms through which gut metabolites regulate these signaling pathways and promote protective immunity.

Fiber-rich diets support SCFA production and maintenance of a higher level of SCFAs in the circulation and intestinal tract^23,56^. In one study, both a high-fiber diet and butyrate feeding in drinking water prolonged survival and ameliorated inflammation during an influenza virus infection. The regulatory actions of SCFA were associated with dampened neutrophil influx and increased the number of patrolling monocytes in target organs. Myelopoiesis of beneficial monocytes was facilitated in the SCFA-enriched condition. The authors concluded that SCFAs predispose M2 macrophage differentiation and production of regulatory cytokines such as IL-10^23^.

Gut microbial composition is closely related to SCFA production and fiber-rich diets contribute to distinct microbial communities. In mouse models, the proportion of *Bacteroidetes* and *Firmicutes* were altered by SCFA or a fiber-rich diet, although the diversity or microbial richness were unchanged. The significance of the microbial community is well studied for effector immune cell induction^23,39^. The importance of gut microbial community has been also observed in several avian models. In one study, the nephrotoxic variant of avian coronavirus altered microbiota composition and thickened the crypt depth^38^. In another study, elimination of the gut microbiota with an antibiotic cocktail induced changes in the microbial composition as well as decreases in the intestinal T cell population and their production of IL-10 and IFN-γ. In that study, SCFA treatment restored the suppressed T cell response^57^. SCFAs stimulate intestinal epithelial cells to produce the chemokines needed for mucosal immunity through GPR activation and HDAC inhibition^58^. Although distinct changes in the microbial community were not observed in our study, probably because SCFA did not reach the intestinal lumen sufficiently, our findings suggest that SCFA administration affected IELs through the interaction with gut metabolites, secretion of effector molecules, and changes in intracellular signaling pathways.

To summarize, we investigated whether SCFAs have beneficial effects against an acute viral infection in chickens. Oral SCFA administration expanded the myeloid and lymphocyte cell populations in multiple organs and facilitated the expression of functional immune molecules that support balanced immunity. Given the limited availability of reagents for studying chickens, our data are not as comprehensive as in mouse studies. However, to our knowledge, this is the first study to examine the immune-regulating actions of major gut metabolites such as SCFAs during avian viral infection. Our data will contribute to further research in this area as well as provide the basis for preventive strategies in the poultry industry.

## Materials and methods

### Virus

The FAdV-4 virus was isolated from the 45-week-old layers with FAdV-4 induced HHS in a commercial specific pathogen free (SPF) chicken flock (Yeoju-city, Gyeonggi province, Korea). The virus was passaged 10 times in Leghorn male hepatoma (LMH) cells (ATCC, USA) and the cells were maintained in Waymouth’s medium (Gibco, USA) supplemented with 1% antibiotics-antimycotics (Gibco, USA).

### Animals

Thirty 1-day-old SPF chickens were randomly assigned to two treatment groups: control and SCFA-supplemented group (SCFA group). Chickens in the SCFA group were provided with drinking water containing sodium acetate (80 mM), sodium propionate (10 mM) and sodium butyrate (20 mM) (Sigma-Aldrich, USA)^57^. The water containing SCFA was filtered and adjusted to pH 7.4. Chickens in the control group were provided with the same volume of normal (not supplemented) water. Water was provided ad libitum. After 72 h, 10 out of 15 chickens (4 days old) in normal water-fed group and another 10 out of 15 chickens SCFA-fed group were injected intramuscularly with 0.1 ml FAdV-4 (10^5^ TCID_50_/ml) and the remaining ten chickens (5 chickens per group) in those two groups were injected with the same volume of PBS (Gibco, USA). At 2 days after injection, the chickens were euthanized with sequential CO_2_ asphyxiation and cervical dislocation in advance to tissue collection. For survival rate, 6 ~ 18 chickens were included. Six animals in uninfected groups (3 chickens in normal water-fed group and 3 chickens SCFA-fed group) and 18 animals in infected groups (9 chickens in FAdV group and 9 chickens in SCFA + FAdV group) from two individual experiments were prepared. The animal work was carried out in compliance with the ARRIVE guidelines and approved by the Institutional Animal Care and Use Committee of Kangwon National University (No. KW-210401-1). All experiments were performed in accordance with relevant guidelines and regulations.

### Cell preparation and histology

The livers, spleens and intestines were collected from each group. Pieces of liver and mucosal contents from intestinal tissues were digested with 4 ml RPMI medium containing collagenase type IV (Worthington Biochemical, USA) for 30 min in at 37 °C. The digested tissues were homogenized in a 40 µm cell strainer, and hepatocytes and intestinal epithelial lymphocytes (IELs) were isolated using the Percoll gradient procedure. After spleen homogenization, erythrocytes were lysed with RBC Lysis Buffer for 2 min and the homogenate was washed. The isolated cells were centrifuged briefly and suspended in complete RPMI containing 10% FBS (Corning, USA), 100 U/mL Penicillin and 100 mg/mL streptomycin (Sigma-Aldrich, USA).

The isolated cells were resuspended in 200 μl of complete RPMI (1 × 10^6^ cells/ml) and centrifuged at 1500 rpm for 3 min using Cytospin 4 Centrifuge (Thermo Fisher, USA). The cytospin preparations were stained with Diff-Quik solution (Sysmex Corporation, Japan).

The livers were collected from the each group and fixed in 10% formalin. The samples were embedded in paraffin blocks and sectioned into 4–5 µm slices with hematoxylin and eosin staining. Histopathological examination was performed in the livers of three representative animals from two individual experiments.

### RNA extraction and quantitative PCR

Total RNA was extracted from the livers, splenocytes, and IELs using TRIzol reagent (Thermo Fisher, USA). cDNA was synthesized with 1 μg of total RNA using Maxima Reverse Transcriptase (Thermo Fisher, USA) according to the manufacturer’s protocol. Quantitative PCR was performed in a 20 μL reaction mixture using Maxima SYBR Green/ROX qPCR Master Mix (Thermo Fisher, USA). All reactions were performed in duplicate.

The threshold cycle (CT) values were obtained using QuantStudio™ 3 Real-Time PCR System (Thermo Fisher, USA) and the expression level was calculated using the formula ${2}^{-\Delta \Delta CT}$. Relative gene expression levels were normalized by β-actin mRNA level. The procedure was performed following MIQE-guideline and relevant studies^6,21,59^. The genes encoding for immune responses were selected, and primers were designed based on the published genome sequences in NCBI, Primer 3, and BLAST databases (Primer designing tool (nih.gov)) The primer sequences used are listed in Table 2^6,49,60–65^.

**Table 2:** Primer sequences for immune molecules.

Genes	Sequence
B-actin	F: TTGTCCACCGCAAATGCTTC
	R: AAGCCATGCCAATCTCGTCT
TNF-α	F: AGATGGGAAGGGAATGAACC
	R: ACTGGGCGGTCATAGAACAG
IFN-α	F: CATCCTGCTGCTCACGCTCCTTCTG
	R: ATCCTGGACACCAGCAACACCCA
IFN-β	F: CCTCCAGCTCCTTCAGAATACG
	R: ACAGCCTCCTCAACCAGATCCAGC
IFN-γ	F: GATGACTTGCCAGACTTACAAC
	R: TAGGTCCACCGTCAGCTACA
IL-1β	F: ACCCGCTTCATCTTCTACCG
	R: TCAGCGCCCACTTAGCTTG
IL-6	F: CCAGAAATCCCTCCTCGCCAATC
	R: GCCCTCACGGTCTTCTCCATAAAC
IL-8	F: GCTCTGTCGCAAGGTAGGA
	R: TGGCGTCAGCTTCACATCT
IL-10	F: CTGTCACCGCTTCTTCACCT
	R: ATCAGCAGGTACTCCTCGAT
IL-12b	F: CCTGTGGCTCGCACTGATAA
	R: TCTTCGGCAAATGGACAGTA
IL-22	F: CAATGCCCATCAAGCCTGCA
	R: ATGCTGAGGATGTGGCACAG
TLR3	F: TCAGTACATTTGTAACACCCC
	R: GGCGTCATAATCAAACAC
TLR7	F: TCTGGACTTCTCTAACAACA
	R: AATCTCATTCTCATTCATCATC
IL-6R	F: CGCCTGCTGGTGGAAGA
	R: TTCACCCGGCAGACGAATTT
CX3CR1	F: TCCAGAACGATCAAGCACAG
	R: CGGTGTTCAGTTCCACATTG
TGFβ-3	F: GAGTCCGAGTACTACGCCAAAGA
	R: CACGTTAAAGCGGAACACATTG
iNOS2	F: GGACCGAGCTGTTGTAGAGA
	R: AGCAGCTGAGTGATGATCCA

### PCR

The fecal swab and liver tissues (100 mg) were obtained from each group at 2 dpi. The samples were homogenized with 1 ml PBS and centrifuged at 3,000 rpm for 10 min. The 200 μl of supernatant was used for viral gene extraction using Maxwell RSC Viral Total Nucleic Acid Purification Kit (Promega, USA). The FAdV gene was detected with conventional PCR using AccuPower PCR premix (Bioneer, Korea). The PCR performed with the following primers: Hexon A 5′-CAARTTCAGRCAGACGGT-3′, and Hexon B 5′- CAARTTCAGRCAGACGGT-3’ (product size 897 bp) as described in a previous study^66^.

### Flow cytometry

Cells isolated from the spleen, liver, and intestinal epithelium were stained with LIVE/DEAD Fixable Aqua Dead Cell Stain kit (L3457; Thermo Fisher, USA) and the following fluorescent antibodies to chicken CD3 (CT-3, Southern Biotech, USA), CD4 (CT-4, Southern Biotech, USA), CD8α (CT-8, Southern Biotech, USA), CD44 (AV6, Southern Biotech, USA), TCRγδ (TCR-1, Southern Biotech, USA), monocytes/macrophages (KUL01, Southern Biotech, USA), MHCII (2G11, Southern Biotech, USA), Bu-1 (AV20, Southern Biotech, USA), and CD45 (LT-40, Southern Biotech, USA). Stained cells were analyzed using a CytoFLEX flow cytometry and the data were analyzed using CytExpert Software (both from Beckman Coulter, USA).

### Microbial analysis

Fecal materials were collected from the cecum 2 days after infection. Genomic DNA was extracted from the cecal contents using a QIAamp DNA Stool Mini Kit (Qiagen Inc, USA) according to the manufacturer’s protocol. Quantitative PCR was then performed using the primers listed in Table 3. The relative gene expression levels were normalized by the total bacterial mRNA levels.

**Table 3:** Primers for microbial analysis.

Genes	Sequence
Total bacteria	F: CGTGCCAGCCGCGGTAATACG
	R:GGGTTGCGCTCGTTGCGGGACTTAACCCAACAT
Actinobacteria	F: TACGGCCGCAAGGCTA
	R: TCRTCCCCACCTTCCTCCG
Bacteroidetes	F: CRAACAGGATTAGATACCCT
	R: GGTAAGGTTCCTCGCGTAT
Enterobacteriaceae	F: ATGTTACAACCAAAGCGTACA
	R: TTACCYTGACGCTTAACTGC
Firmicutes	F: TGAAACTYAAAGGAATTGACG
	R: ACCATGCACCACCTGTC

### Statistics

The statistical significance of the flow cytometry was determined using Tukey’s multiple comparison with one way ANOVA. Student’s unpaired t-test was used to assess the significant differences in the quantitative PCR analysis. *P* values less than 0.05 were considered as statistically significant.

### Supplementary Information

Supplementary Information.

## Data Availability

All data used in this study available from the corresponding author on reasonable request.
