# Supplementary material for: Short chain fatty acids facilitate protective immunity by macrophages and T cells during acute fowl adenovirus-4 infection
Source: Sci Rep. 2023 Oct 21;13:17999. doi: 10.1038/s41598-023-45340-8 (PMC10590440; doi:10.1038/s41598-023-45340-8)
Supplement: Supplementary file 1 — Supplementary Information. [file 41598_2023_45340_MOESM1_ESM.docx]

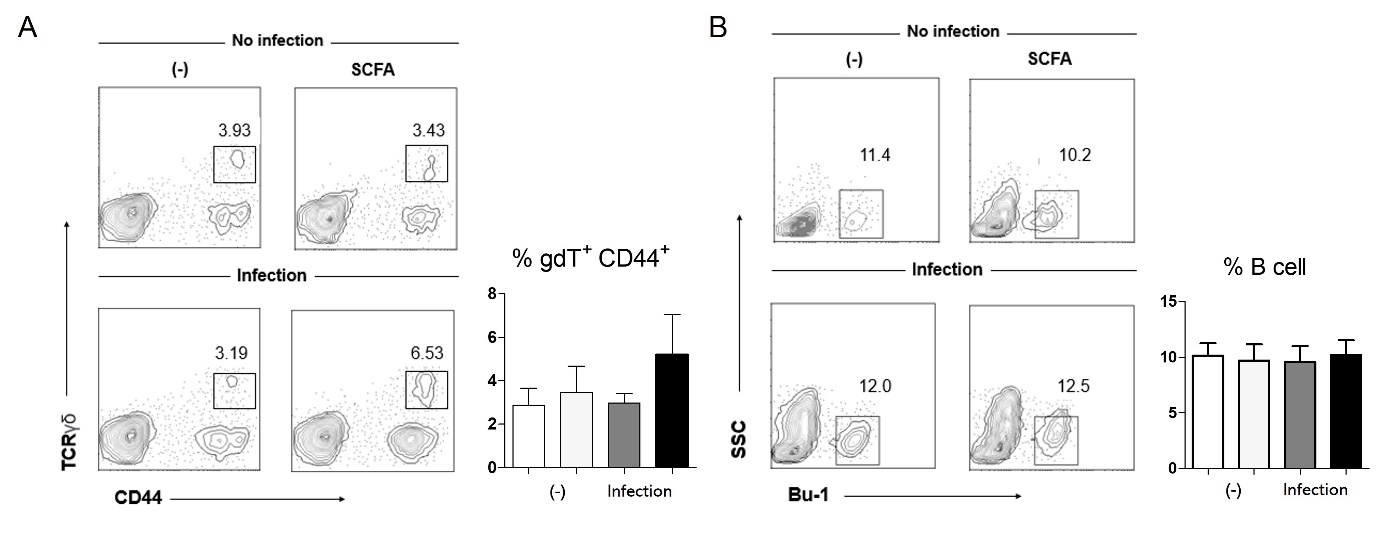


**Supplementary Figure 1. Effects of SCFAs on γδ-T cell and B cell** **in the liver during FAdV-4 infection.** The frequencies of γδ-T cell and B cell in liver were determined using flow cytometry at 2 dpi. (A) γδ-T cells were examined. Representative plots are shown. (B) Frequencies of B cells were calculated and representative plots are shown. Cells were obtained from 3~4 chickens per group. Two independent experiments were performed, and pooled data are shown. Significant differences were identified using unpaired t test. (p<0.05).


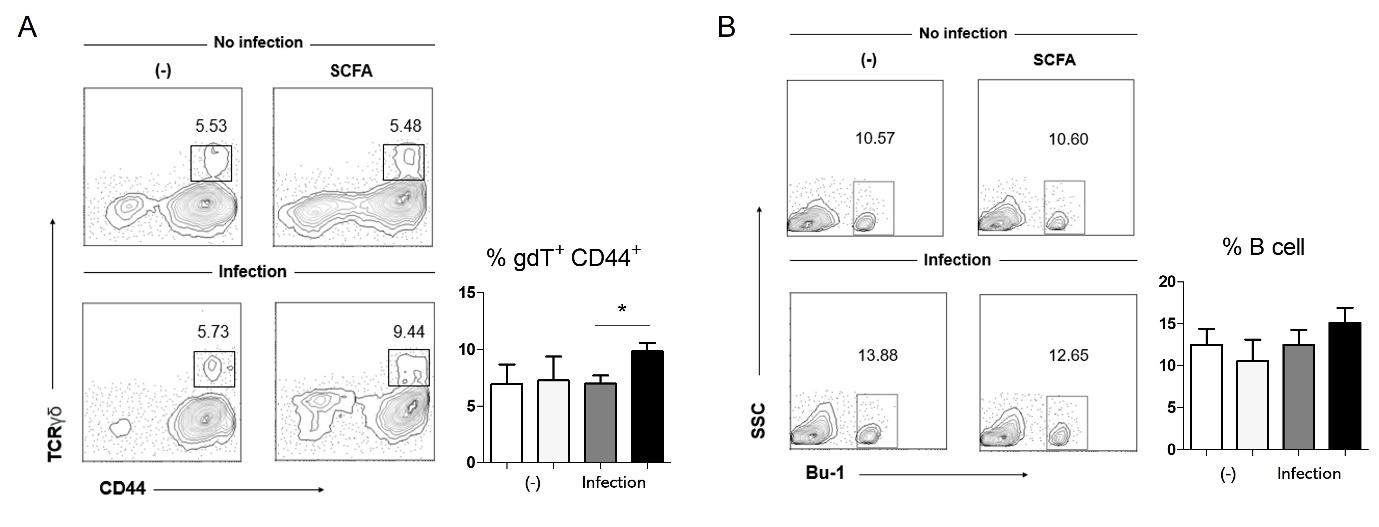


**Supplementary Figure 2. Effects of SCFAs on γδ-T cell and B cell** **in the spleen during FAdV-4 infection.** The frequencies of γδ-T cell and B cell in spleen were determined using flow cytometry at 2 dpi. (A) γδ-T cells were examined. Representative plots are shown. (B) Frequencies of B cells were calculated and representative plots are shown. Cells were obtained from 3~4 chickens per group. Three independent experiments were repeated, and pooled data are shown. Significant differences were identified using unpaired t test. (p<0.05).


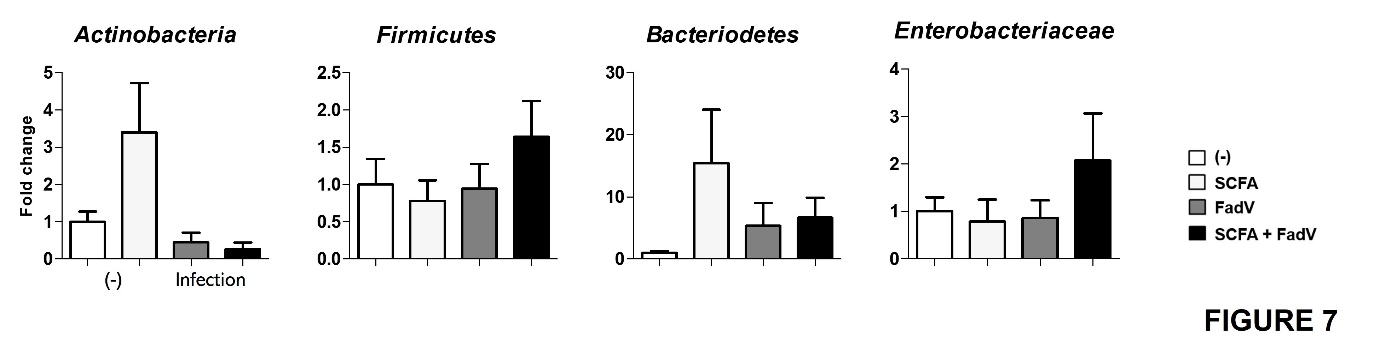


**Supplementary Figure 3. Regulation of gut microbial composition by SCFA treatment and FAdV-4 infection.** Bacterial genomic DNA in cecal material was extracted at 2 dpi, and DNA was analyzed by qPCR. The relative expression of bacterial gene was compared with the total bacterial expression level. Data are presented as the fold change relative to the naïve group. Fecal samples were collected from 1~3 chickens for each group, and the experiments were performed at least three times. qPCR was conducted in duplicate. Significant differences were identified using unpaired t test (*p<0.05).
